# Trends and Challenges in Neuroengineering: Toward “Intelligent” Neuroprostheses through Brain-“Brain Inspired Systems” Communication

**DOI:** 10.3389/fnins.2016.00438

**Published:** 2016-09-23

**Authors:** Stefano Vassanelli, Mufti Mahmud

**Affiliations:** NeuroChip Laboratory, Department of Biomedical Sciences, University of PadovaPadova, Italy

**Keywords:** neuroengineering, biohybrid systems, neurobiohybrid systems, neuromimetic systems, brain-chip interfaces, brain machine interfaces, neurorehabilitation, artificial sensory organs

## Abstract

Future technologies aiming at restoring and enhancing organs function will intimately rely on near-physiological and energy-efficient communication between living and artificial biomimetic systems. Interfacing brain-inspired devices with the real brain is at the forefront of such emerging field, with the term “neurobiohybrids” indicating all those systems where such interaction is established. We argue that achieving a “high-level” communication and functional synergy between natural and artificial neuronal networks *in vivo*, will allow the development of a heterogeneous world of neurobiohybrids, which will include “living robots” but will also embrace “intelligent” neuroprostheses for augmentation of brain function. The societal and economical impact of intelligent neuroprostheses is likely to be potentially strong, as they will offer novel therapeutic perspectives for a number of diseases, and going beyond classical pharmaceutical schemes. However, they will unavoidably raise fundamental ethical questions on the intermingling between man and machine and more specifically, on how deeply it should be allowed that brain processing is affected by implanted “intelligent” artificial systems. Following this perspective, we provide the reader with insights on ongoing developments and trends in the field of neurobiohybrids. We address the topic also from a “community building” perspective, showing through a quantitative bibliographic analysis, how scientists working on the engineering of brain-inspired devices and brain-machine interfaces are increasing their interactions. We foresee that such trend preludes to a formidable technological and scientific revolution in brain-machine communication and to the opening of new avenues for restoring or even augmenting brain function for therapeutic purposes.

## Introduction to *Neurobiohybrids*

### An overview on biohybrids

The research field of biohybrid systems (or biohybrids) is capturing increasing interest across various scientific communities. The deepening of our knowledge on the physiology of living organisms –down to the cellular and molecular level– and the progress in the engineering of miniaturized interfaces between living and artificial systems, are driving research toward the creation of biohybrids where boundaries between living beings and man-made artifacts are collapsed. Classically, individual scientific communities are approaching this type of research from a different perspective. For example, within the “*robotics*” and “*biomimetics*” community, biohybrid systems are generally considered as an opportunity to exploit the unique characteristics of biological systems or their components, refined over millions of years of natural evolution, in order to solve complex or critical problems hampering artificial systems performance (Ricotti and Menciassi, 2012; Wilson et al., 2015). In this “learning from nature” endeavor, biological systems are seen as a source of inspiration for innovative solutions, toward a “soft” and “wet” robotics or “living” systems/technologies characterized by self-organization, evolvability, adaptability, and robustness (Eiben et al., 2012). Thus, biohybrid systems come here into play as workbenches where to experiment how to build “living” artificial systems.

**Figure 1:**
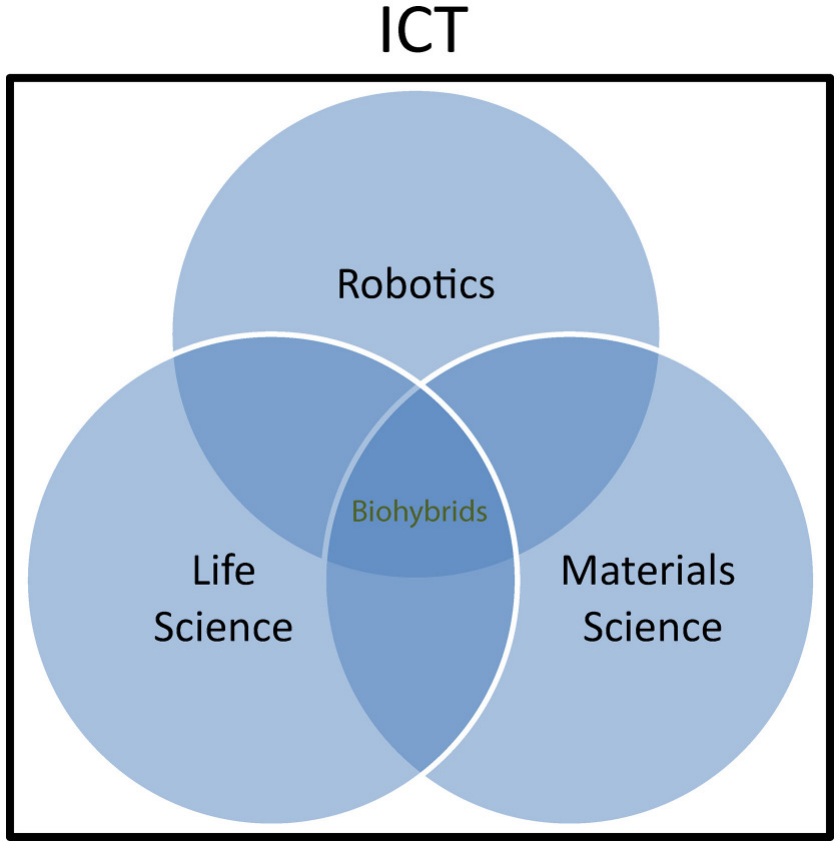
**Biohybrids as interdisciplinary research field**. Within Information and Communication Technologies (ICT), Robotics, Life science, and Materials science communities are involved in biohybrids research. Scientific cooperation and interaction within the three communities is growing rapidly, although the involvement of the different actors varies by extend and typology depending on the specific research topics.

On the other hand, biohybrids are seen by the “*life science*” community as useful tools to explore the physiology of living organisms or even as therapeutic tools. Whenever new and more advanced ways of communication with the living matter are developed, new opportunities arise to extend our capability to measure biological parameters that are relevant for understanding physiological mechanisms. Furthermore, building artificial artifacts emulating physiological operations and interacting with natural systems is a way to assess biological working hypotheses through a “reverse engineering” and reductionist approach. Biohybrids bear a huge and yet unexplored potential also for medical application, through the embodiment of natural “intelligence” and material properties in diagnostic and therapeutic tools. Neuroprostheses (Hochberg et al., 2006) and bioelectronics medicines (Birmingham et al., 2014) represent a typical example and it is easy to assume that much effort will be deployed in the future to implement artificial devices with near-physiological characteristics and communication properties for restoring function in humans. Finally, the “*materials science and engineering*” community is active in investigating fundamentals of interfacing between living matter and inorganic material. This work goes at the root of biohybrid research and has an increasing impact on other classical disciplines, including chemistry and biology. For example, thanks also to the availability of a synthetic toolbox to conjugate biomolecules and synthetic polymers in a controlled fashion, combining biomolecules, and synthetic polymers into a new class of versatile biohybrid materials following a “click” chemistry methodology has gained much interest in recent years (Dirks et al., 2007). The concept of interdisciplinary coverage of biohybrids research is sketched in Figure 1.

### Biohybrid: a working definition

A common definition of biohybrid system that accepted by the scientific community is still missing. Thus, we propose a working definition to be shared with researchers interested to the field and eventually to be further refined in the future. As the term *biohybrids* encompasses a heterogeneous “melting pot” of systems spanning a range from the macro- to the nanoscale, we propose a comprehensive working definition, which highlights the importance of information exchange between living and artificial entities and its processing.

*Biohybrid: a working definition.* A *biohybrid*, is a system formed by at least one natural and at least one artificial entity that establish close physical interactions at the molecular, cellular, or systems level, eventually leading to information flow and processing in one or both directions.

### Neurobiohybrids

**Figure 2:**
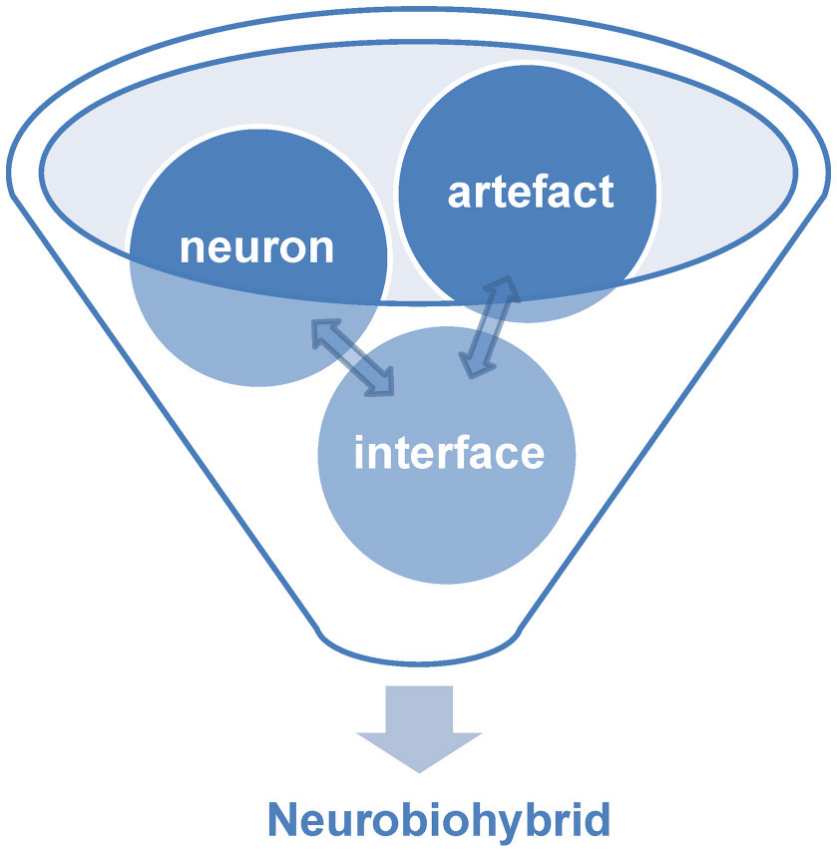
**Components of neurobiohybrid**. A Neurobiohybrid is formed by three fundamental components: neuron, artifact and an interface, the latter with the function of establishing a uni- or bi-directional communication between the two.

Within the world of biohybrids, neurobiohybrids are those where the natural component is represented by neurons. They can be present in the form of individual cells or networks, and either *in vitro* (i.e., cell culture or brain slice preparations) or *in vivo* (i.e., within the nervous system of a living animal). In general, within a neurobiohybrid, the artificial part will be composed by two functional units: (1) a device (or more devices) that have to establish the communication with neurons; (2) an interface, which mediates the physical interaction between neuron(s) and device(s), allowing the transfer of information between biological and artificial components, either in one or both directions, and its processing (Figure 2).

**Figure 3:**
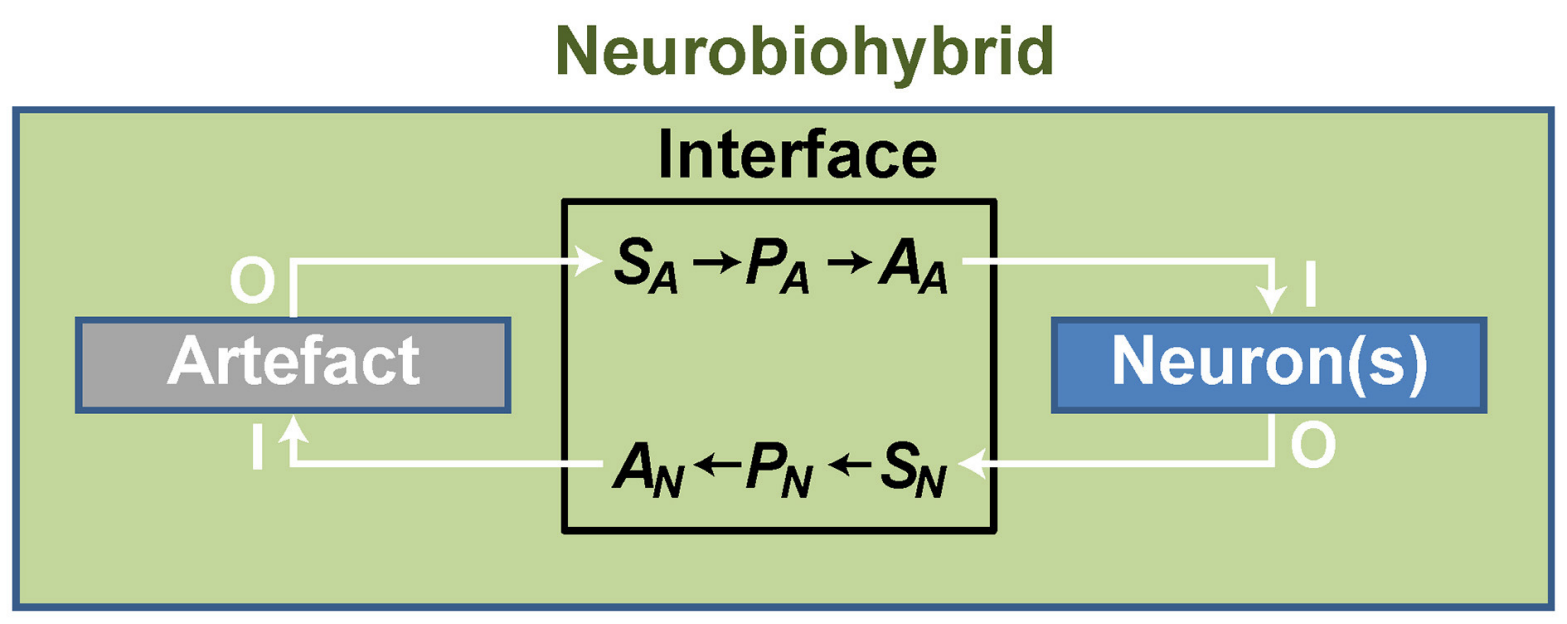
**General scheme of a neurobiohybrid**. Artificial and natural components, i.e., artifact and neuron(s) respectively, communicate through a bidirectional interface. Here, signals are detected and converted by transducers, the sensor elements **S**_**A**_ and **S**_**N**_, processed by processing units, **P**_**A**_ and **P**_**N**_, and fed through actuators, **A**_**A**_ and **A**_**N**_.

In practice, artificial devices, such as computers or bionic neuroprostheses, are communicating with neurons through energy exchange occurring in one or both directions and forming a new system acting as a whole. Whatever the approach adopted to create the neurobiohybrid system, a crucial component is represented by the interface that must include several fundamental elements to operate. First of all, in case of neuron-to-device communication, a sensor is needed, **S**_**N**_, transducing neuronal signals (Figure 3); second, a processing unit, **P**_**N**_, elaborates transduced signals; third, another transducer, the actuator **A**_**N**_, transforms the output signals from the processing unit into signals suitable to control the device. Similarly, in the opposite direction, signals from the device control the neuronal response through a chain formed by a sensor (**S**_**A**_), a processing unit (**P**_**A**_), and an actuator (**A**_**A**_) (Figure 3).

We must clarify how this general description of the interface is wide-ranging:

- Bidirectional communication is not an absolute requirement, as unidirectional communication is sufficient to establish a neurobiohybrid;
- Communication can occur through any type of energy conveying information (electromagnetic, chemical, mechanical). Thus, any possible mechanism allowing information exchange and processing within the neurobiohybrid is included;
- “Processing” is here intended as “operating on time-varying physical quantities.” As such, the term does not solely comprise the more conventional digital or analog signal processing, but rather any type of processing that can be operated by any type of suitable processing unit, e.g., from single molecules to electronic computing architectures.

According to this introduction and the definition of biohybrid provided above, we propose a working definition of neurobiohybrid.

*Neurobiohybrid: working definition.* A *neurobiohybrid* is a system formed by the combination of at least one neuron as natural entity and at least one device as artificial entity. To form a neurobiohybrid system, neuron(s), and device(s) establish physical interactions through an interface at the molecular, cellular, or systems level, eventually leading to information transfer and processing in one or both directions.

Noteworthy, according to such definition, all those implementations that are commonly known as brain-computer-interfaces (BCI) fall under the neurobiohybrids umbrella. Included are also those therapeutic hybrid systems working through stimulation and/or recording of the central or peripheral nervous system, and that are relying on appropriate interfacing for information transfer and processing. Typical examples based on “invasive” interfacing are deep brain stimulation (DBS, McConnell et al., 2016), neuroprosthetic limbs (e.g., Micera, 2016), cochlear implants (Roche and Hansen, 2015; Sato et al., 2016), or artificial retinas (e.g., Zeck, 2016). Interestingly, also “non-invasive” interfacing approaches such as functional electrical stimulation (FES) (for a review see Peckham and Knutson, 2005) or transcranial current stimulation (TCS, Ruffini et al., 2013) should be considered as part of the neurobiohybrids family. Given the obvious relevance of neurobiohybrids in computer science, basic neuroscience, and therapy of neurological disorders, it is no surprise that funding agencies are devoting resources to attack major challenges in the field (e.g., Miranda et al., 2015). However, we believe that, among major challenges, learning how to create functional hybrids between biological neural networks and neuromimetic architectures emulating their processing capabilities has an immense potential, particularly in the perspective of restoring or even augmenting brain function.

## *Neurobiohybrids*: state-of-the-art, current trends, and challenges

In this section, a brief overview on the state-of-the-art and current trends in neurobiohybrids research is given, with particular emphasis on recent advances originating from the convergence of novel neurotechnologies and neuromimetics research. Specifically, we focus on those paving the way to “intelligent” neuroprosthetics for restoring and augmenting brain function in living animals. We identify key scientific and technological challenges as also pointed out by Thibeault (2014), and briefly discuss opportunities and threats for the development of the neurobiohybrids research community.

### Neurobiohybrids: state-of-the-art and main trends

We recognize in the “dynamic clamp” technique the first fundamental leap into research on neurobiohybrids. Here, although in *in vitro* conditions, artificial neuromimetic systems are physically and functionally coupled to biological neurons with mutual information exchange in a clear manner. The dynamic clamp relies on a closed-loop control over the neuronal intracellular potential and membrane conductances, the controller being an elementary analog or software-based neuronal counterpart (Sharp et al., 1993; Prinz et al., 2004). Although ground-breaking, and despite significant improvements from the time of its introduction, this method is not suited for long-term and large-scale network implementations, as it is intrinsically limited by the interfacing through intracellular electrodes.

Brain processing, instead, deeply relies on neuronal circuits. Therefore, multi-site—and minimally invasive—techniques are necessary, allowing to interface many neurons at once within the neurobiohybrid. Attempts have been made to create network-based neurobiohybrids and in the first instance, in *in vitro* systems. For example, metal multi-electrode arrays (MEA, for a historical review of MEA, see Pine, 2006) were used to interface networks of dissociated neurons to a robot actuator where the processing was taken over by software-based spike encoding/decoding algorithms (Novellino et al., 2007). In addition, parallel progress made on neural interfaces for large-scale high-resolution multi-site recording techniques and neuromimetic nanodevices and architectures, have opened up new avenues. Recording and stimulating *in vitro* with large and dense arrays of voltage transducers (Hutzler et al., 2006; Hierlemann et al., 2011; Ferrea et al., 2012; Lewandowska et al., 2016) and optical imaging techniques (Chemla and Chavane, 2010; Tian et al., 2012) allow the gathering of spiking or sub-threshold signaling events from large neuronal networks.

Studies *in vivo*, instead, have led to implant-based BCIs and brain-machine-interfaces (BMIs) taking advantage of advanced multi-site neural interfaces and real-time software-based processing for neuroprosthetic applications. Numerous examples can be found in literature, from basic research to translational medicine, and ranging from rodents (e.g., Shobe et al., 2015), to non-human primates (Zhang et al., 2016) and even to human subjects (Hochberg et al., 2006). Although constrained in terms of number of recording/stimulation sites in comparison to their *in vitro* counterpart (Csicsvari et al., 2003; Berényi et al., 2014; Vassanelli, 2014; Schroder et al., 2015), their importance for investigating neurons in an intact brain (Buzsáki et al., 2012), or as interfaces for brain-machine communication and neuroprosthetics is well recognized (Nicolelis and Lebedev, 2009; Lebedev and Nicolelis, 2011).

However, a real paradigm shift toward “intelligent” neuroprosthetics and brain augmentation can be expected from the creation of neurobiohybrids where such brain interfaces are functionally coupled to neuromimetic devices and architectures emulating brain circuits (Thibeault, 2014). In fact, in our view, similarly to what happens for cardiac pacemakers (see Miller et al., 2015; Seriwala et al., 2016) or more “classical” prostheses (e.g., orthopedic prostheses, see Goldfarb et al., 2013; Ortiz-Catalan et al., 2014; Raspopovic et al., 2014; Vujaklija et al., 2016), the challenge is to engineer artificial neuronal systems emulating as closely as possible their natural counterpart and interfacing them efficiently to the native organ to restore (or to augment) function. Recent neuromorphic architectures based on very large scale integration (VLSI) technology (Indiveri et al., 2011; Qiao et al., 2015) and the discovery of physical components with synaptic-like plasticity properties such as memristors (Strukov et al., 2008) or carbon nanotubes based circuits (Joshi et al., 2011), have set the foundations for developing such novel generation of neurobiohybrids.

Large improvements and innovations are unquestionably necessary to achieve effective communication between natural and artificial neuronal networks. Two-way (recording and stimulation), high-resolution (down to micrometers), and large-scale (hundreds to thousands of neurons) interfacing is still beyond reach. Particularly, in this context, techniques for large-scale and high-density stimulation (also known as microstimulation) are lagging behind expectations. Although optogenetic platforms may be suitable candidates (Dugue et al., 2012; Buzsáki et al., 2015; Grosenick et al., 2015; Newman et al., 2015), other means of stimulation not requiring neuronal transfection with biological agents are to be taken into account for real clinical applications (e.g., via tuneable and field-shaped electrical stimulation or localized neurotransmitters detection and release).

### Scientific and technological challenges

#### Transducers (sensing and actuating)

As hinted above, the development of novel sensing and actuating probes is expected to play a fundamental role in the neurobiohybrids field toward application in neuroprosthetics. A wide range of probes with different materials, design, and fabrication processes, and interfacing principles have been developed and reported earlier targeting specific research needs (for reviews see Wheeler and Nam, 2011; Spira and Hai, 2013; Vassanelli, 2014; Vidu et al., 2014; Angle et al., 2015; Fekete, 2015; Giocomo, 2015; Ruther and Paul, 2015; Lee et al., 2016; Patil and Thakor, 2016; Pisanello et al., 2016; Prodanov and Delbeke, 2016). Brain-chip interfaces are among most promising strategies to support such development (Vassanelli et al., 2012) as semiconductor technology allows for integration into a single millimeter scale device of a large number (hundreds to thousands) of microtransducers for recording and stimulation of neuronal signals.

Concerning interfaces based on electrical signaling between neurons and chips (those that are most developed so far), two fundamental approaches exist depending on the nature of the transducer:

1. Neural interfaces based on metal microelectrodes were developed first, and are now available in the form of 2D or 3D arrays that can be implanted in the brain or following a different interfacing philosophy, connected to peripheral nerves (Rutten, 2002; Wise et al., 2004; Stieglitz et al., 2014). The neuroelectronic interface is established when neuron and microelectrode are “close enough,” allowing signal detection (from neuron to microelectrode) or stimulation (from microelectrode to neuron). This condition can be met both *in vitro* and *in vivo*, although under different biophysical bases. In the *in vitro* case, neurons are typically cultured on the chip surface where their membrane come into close contact with microelectrodes (i.e., typically in the tens of nanometers range) by adhering to the solid chip substrate (Braun and Fromherz, 2004). Though the original brain network topology is lost, the dissociated neurons reconnect and form a more random-like network (Haider and McCormick, 2009; Kwan and Dan, 2012). Recent technological advances allowed the development of large-scale high-density metal electrode arrays (MEA) for high-resolution recording of such neuronal networks in culture (Eversmann et al., 2011; Maccione et al., 2012; Muller et al., 2015). When a MEA, instead, is implanted in the nervous system (i.e., brain or spinal cord), transducers and neurons are more separated than in *in vitro* conditions, as cell adhesion is not governing neuro-chip interaction in this context. Also, in case of chronic implants, damaged tissue first, and gliosis afterwards are commonly building a separation layer between transducers and neurons. Thus, a volume conductor of tissue surrounds the interface and ionic currents and voltage gradients developing within it are governing recording and stimulation of neurons (Mitzdorf, 1985; Gold et al., 2006; Anastassiou et al., 2010). Other types of microstructured metal electrode-based interfaces exist, as regenerating sieves (Lago et al., 2005) and cone-in-growth electrodes (Rutten, 2002). In addition, alternative to brain implantation, microfabricated cuff or intrafascicular electrodes can be used to interface peripheral nerves (Mailley et al., 2004). Whatever the site of implantation, owing to electrochemical features of the electrolyte-metal electrode interface, faradaic currents are likely to occur, particularly when relatively large potentials come into play, i.e., during stimulation (Vassanelli, 2014).
2. The second fundamental strategy for neuron-chip interfacing aims to solve this problem by using oxide-insulated semiconductor or metal-semiconductor transducers to generate a capacitive coupling with neurons (Fromherz, 2006; Eickenscheidt et al., 2012). The approach has the advantage of relying on non-faradaic currents, at least within wider voltage ranges. Basing on this approach, very high-resolution CMOS chips have been developed for electrical imaging of neurons *in vitro* (Hutzler et al., 2006) and more recently, for *in vivo* applications (Felderer and Fromherz, 2011; Schroder et al., 2015). Noteworthy, as excitatory and inhibitory neurons are expected to respond differently to appropriately selected electrical stimuli (Mahmud and Vassanelli, 2016a), it will be strategically important to achieve a high degree of control over the electrolyte-microelectrode interface to achieve a finely tuned stimulation of neurons.

Finally, it is worth to mention that electrical neural interfaces will be improved also by clever use of novel materials. For example, read-out of neuronal activity from the mammalian brain *in vivo* was achieved by means of injectable free-standing mesh electronics (Liu et al., 2015), thus potentially minimizing tissue damage and reaction and reaching and unprecedented level of intermingling between neural tissue and electronics.

With the advent of optogenetic stimulation (Dugue et al., 2012) new hybrid optoelectronic interfaces are emerging (Park et al., 2011; Armstrong et al., 2013; Wu et al., 2013; Pashaie et al., 2015). With respect to electric stimulation (Mukaino et al., 2014; Tabot et al., 2015), optogenetics offers basically two potential advantages: (i) neuronal type specificity and (ii) the possibility to inhibit and not only to excite target neurons. It is therefore easy to foresee that a considerable amount of work will be deployed to exploit these characteristics in neurobiohybrids for controlling neuronal circuit activities within a closed-loop at cellular rsolution (Packer et al., 2015).

Although invasive interfaces are the most suitable to enable a reliable and high-resolution communication with the brain, several sorts of non-invasive brain-machine interfaces are also available (Waldert, 2016). They can be indeed included within the neurobiohybrids scheme, albeit based on unidirectional communication. They include for example EEG based platforms (Friehs et al., 2004; Norton et al., 2015), but also recent developments on fMRI for real time brain-machine interfacing (Weiskopf et al., 2007; Lee et al., 2009; Ruiz et al., 2014). On the other hand, functional electrical stimulation (FES) or transcranial current (TCS) (Ali et al., 2013) or magnetic (TMS) (Camprodon, 2016) stimulation approaches are to be included as they allow for machine-to-brain communication. Although limited in spatiotemporal resolution, non-invasive interfaces offer undoubtful advantages in terms of clinical application (Ortiz-Rosario and Adeli, 2013). “Hybrid” less-invasive solutions such as high-resolution electrocorticography (ECoG), represent an interesting compromise when cortical areas are to be interfaced for recording and perhaps, stimulation (Girardi et al., 2011; Vassanelli et al., 2012; Berényi et al., 2014; Khodagholy et al., 2015) because of their limited invasiveness with respect to in-brain implants (Pei et al., 2011; Matsushita et al., 2013).

#### Processing unit

Fast processing of neuronal signals is essential for real-time performance in neurobiohybrids. When dealing with one or a few neurons, this is achievable also on the basis of conventional software or analog circuits. However, when dealing with larger networks, high-performance approaches must be considered for simultaneous real-time processing of multiple neuronal signals. A detailed analysis of state-of-the art signal processing tools for brain-machine interfaces goes beyond the scope of this article and is available elsewhere (see, for example, Krusienski et al., 2011; Mahmud et al., 2012, 2014; Mahmud and Vassanelli, 2016b). However, we think that among parallel computing architectures speeding up processing times, “intelligent” neuromorphic analog processors based on artificial neuromorphic neural networks (see for example Qiao et al., 2015) will play a major role in the next generation of neurobiohybrids. As a matter of fact and similarly to other prostheses, in neuroprosthetics the ideal goal is replacing neuronal networks that have undergone injury or degeneration with artificial circuits emulating as closely as possible native functional features. Artificial neurons owning functional properties similar to their natural counterparts such as firing behavior and plasticity-based synaptic integration, will be an ideal replacement or rehabilitation support for injured or degenerating neuronal circuits in neuroprosthetics.

#### Dynamic clamp

The dynamic clamp technique offers a prototypic example of such vision, where an artificial brain-inspired computing system drives the excitability of a living neuron establishing a real-time closed-loop control within a neurobiohybrid system. Beginning in the early 90s, researchers started to investigate the interaction of living nerve cells in culture with model neurons in order to understand and emulate the behavior of neural networks (Yarom, 1991; Le Masson et al., 2002). The interface connection between living and model neurons was based on intracellular electrodes, in fact obtaining a two-way artificial-natural communication within a neurobiohybrid. Due to intrinsic limitations of intracellular electrode techniques, such neurobiohybrid setup was limited to only one, or a few, biological neurons. On the other hand, having access to the intracellular potential, it had the advantage of providing high sensitivity for detecting and eliciting neuronal signals. From the neurobiohybrid perspective (see Section Neurobiohybrids), the “device” was here represented by a biomimetic artificial neuron. We may refer to this type of neurobiohybrid, where the interfaced device is a biomimetic artificial neuron, as a Neuron-Neuron Hybrid (NNH). NNH gained interest as a mean to correlate experimental and modeling studies through a sort of reverse-engineering approach, taking advantage of biological neurons to validate their emulators as well as working hypotheses on operational properties of neuronal circuits. Biomimetic neurons and their networks can be digital or analog. Elementary NNHs and their networks have been investigated through the dynamic clamp (Sharp et al., 1993; Prinz, 2004; Yang et al., 2015), which was used to monitor the membrane potential of living neurons and via numerical simulation of model neurons and synapses on a computer, to inject synaptic currents into the living neurons in real-time, as if they were “synaptically” connected to the model neurons. Alternatively, the dynamic clamp could be used to “insert” artificial membrane conductances into living neurons embedded in a network, thus exploring the role of intrinsic conductances in shaping the network's output.

#### From single neurons to networks

In hybrid NNHs with analog model neurons and synapses, a specially designed microelectronic circuit constitutes the artificial part of the network. Such hardware model neurons and synapses can be connected to living circuits through electrodes, creating a hybrid circuit that consists of a biological network and a dedicated “neuromorphic” (or neuromimetic) silicon chip. With the development of multi-electrode approaches (Rutten, 2002), pioneering work has first succeeded to interface, through dedicated software, cultured neurons and robots, a step toward the creation of “autonomous intelligent biohybrid systems” (Novellino et al., 2007). In other examples of neurobiohybrid network applications, Nowotny et al. (2003) used a hybrid circuit with an Aplysia neuron to show that spike-timing dependent plasticity (STDP) enhances synchronization in neural networks, while Manor and Nadim (2001) demonstrated that synaptic depression in neural networks with recurrent inhibition gives rise to bistability by combining a digital model neuron with a biological pacemaker neuron. In a particular elegant study, Le Masson et al. (2002) reconstructed a thalamocortical circuit by coupling living neurons in the lateral geniculate nucleus to digital and analog model neurons. The researchers showed how feedback inhibition can functionally disconnect the cortex from sensory input in a state reminiscent of sleep, demonstrating the potential of the “Natural-Artificial-Neurohybrid” and/or hybrid NNHs approach in elucidating network function even in large circuits. Overall, when examined from a broader perspective, this sort of pioneering investigations on hybrid networks can be also interpreted as a part of a general effort in the search for novel experimental approaches to investigate neural microcircuits and to develop more efficient brain-machine interfaces for neurological therapy and rehabilitation.

#### Artificial neuromorphic neuronal networks

Integration into a unique neurobiohybrid system of large (i.e., tens to hundreds of neurons) neuronal networks is a major challenge to be faced. To this endeavor, Very Large Scale Integrated (VLSI) devices come into play. VLSI devices comprise hybrid analog/digital circuits that implement hardware models of biological systems, using computational principles analogous to the ones used by nervous systems (Indiveri and Horiuchi, 2011; Indiveri, 2015). When implemented in VLSI technology, neuromorphic circuits use, to some extent, similar physics used in neural systems (e.g., they transport majority carriers across the channel of transistors by diffusion processes, very much like neurons transport ions inside or outside cell bodies through their ionic channels). Given the analogies at the single device level, neuromorphic circuits are ideal interfacing circuits to real neurons. Moreover, larger scale neuromorphic networks of spiking neurons share the same physical constraints of their biological counterparts (i.e., noise, temperature dependence, inhomogeneities, etc.). As a consequence, to carry out computation in a robust and reproducible manner, these architectures often have to use similar strategies for maximizing compactness, optimizing robustness to noise, minimizing power consumption, and increasing fault tolerance.

In recent years, an interesting class of neuromorphic devices implementing general-purpose computational architectures based on networks of silicon neurons and synapses emerged (Bartolozzi and Indiveri, 2007; Indiveri et al., 2011; Indiveri and Liu, 2015). Such devices range from reconfigurable arrays of basic integrate and fire neuron models to learning architectures implementing detailed models of spike-based synaptic plasticity. Spike-based plasticity circuits enable these systems to adapt to the statistics of their input signals, to learn and classify complex sequences of spatio-temporal patterns (e.g., arising from visual or auditory signals), and eventually to interact with the user and the environment. Typically, the analog circuits implemented on these devices operate in the *weak-inversion* regime, where current amplitudes are of the order of pico-Amperes and operating time-constants are of the order of milliseconds. This is a crucial characteristic that differentiates this approach with other more conventional full custom analog VLSI approaches for implementing spike-based neural networks. Conventional analog VLSI implementations of spike-based neural networks use circuits biased in the *strong-inversion* region, that produce currents of the order of micro-amperes, so the largest time-constants that can be achieved in practice are at least 1000 times smaller than biological ones. The biologically plausible time constants achieved with the neuromorphic approach are crucial, as they allow seamless interactions with real living networks. Given the types of parallel architectures that can be implemented with these silicon neurons and synapses, processing time does not increase with size, and large networks can be fabricated by (e.g., simply using more silicon real-estate) to match the numbers of recording/stimulating electrodes or real targeted neurons that one would like to interact with.

Consistent with the neuromorphic engineering approach, the strategy used to transmit signals across chip boundaries in these types of systems is inspired from the nervous system: output signals are represented by stereotyped digital pulses (spikes), and the analog nature of the signal is typically encoded in the mean frequency of the neuron's pulse sequence (spike rates). Similarly, input signals are represented by spike trains, conveyed to the chip in the form of asynchronous digital pulses, that stimulate their target synapses on the receiving chip. The circuits that generate the on-chip synaptic currents when stimulated by incoming spikes are slow low-power analog circuits. The circuits that generate and manage these streams of input/output digital pulses are fast asynchronous logic elements based on an emerging new communication standard for neuromorphic chips called the “Address-Event Representation” (AER). This representation is ideal for both implementing real-time interfaces with living networks, as well as for allowing reconfigurability of artificial network topology (e.g., via address-event source-destination lookup tables).

#### Memristive plasticity

An important advancement in the field of biological networks emulation and with great potential in neuroprosthetics is the development of new nanoelectronic elements with synaptic functional properties. Carbon nanotubes (Cellot et al., 2009; Joshi et al., 2011; Fabbro et al., 2013) and particularly, memristors are emerging as a new class of devices that might serve the purpose. Resistive Random Access Memory (ReRAM) cells are nowadays classified as being memristive in nature (Chua, 2011) and have first being conceptually conceived in 1971 by Chua (1971), with the first neuromimetics applications presented at the same time. Since then, the usage of memristors in simulating artificial synapses has started to be explored (Yang et al., 2013; Kim et al., 2015; Niehrster and Thomas, 2015; Thomas et al., 2015). The functional signature of memristors is a pinched hysteresis loop in the current-voltage (i-v) domain when excited by a bipolar periodic stimulus. Such hysteresis is typically noticed for all kind of devices/materials in support of a discharge phenomenon that possess certain inertia, causing the value of a physical property to lag behind changes in the mechanism causing it, and has been common both to large scale (Prodromakis et al., 2012) as well as nanoscale dissipative devices (Strukov et al., 2008). The analogy of memristors and chemical synapses is thus made on the basis that synaptic dynamics depend upon ions flowing through the postsynaptic membrane in a similar fashion that “ionic species” can be displaced within any inorganic barrier. TiO2-based memristor models (Strukov et al., 2008; Prodromakis et al., 2011) hypothesized that solid-state devices comprise a mixture of TiO2 phases, a stoichiometric and a reduced one (TiO2-x), that can facilitate distinct resistive states via controlling the displacement of oxygen vacancies and thus the extent of the two phases. More recently however it was demonstrated that substantial resistive switching is only viable through the formation and annihilation of continuous conductive percolation channels (Shihong et al., 2012) that extend across the whole active region of a device, shorting the top (TE) and bottom (BE) electrodes; no matter what the underlying physical mechanism is.

The development of such emerging nanoscale synaptic-like computation elements may notably benefit the establishment of neuromorphic architectures and neurobiohybrids. This technology adds substantially on computation functionality, due to the rate-dependency of the underlying physical switching mechanisms. At the same time it can facilitate unprecedented complexity due to the capacity of storing and processing spiking events locally. Moreover, the minuscule dimensions and architectural simplicity of solid-state memristor implementations could be successfully exploited to substantially increase the number of cells per unit area, and effectively enhance the system's tolerance to issues stemming from device mismatch and low-yields (Gelencser et al., 2012; Gupta et al., 2016).

### Toward “intelligent” neuroprosthetics

In perspective, neurobiohybrids, and particularly NNHs, will represent the basis for creating advanced and “intelligent” neuroprostheses. Novel generations of neuroprostheses or bioelectronic medicines (BM) acting through electrical stimulation of the central or peripheral nervous system bear a huge potential for therapy of numerous diseases, including neurological disorders, metabolic, and autonomic dysfunctions (Hyam et al., 2012; Afshar et al., 2013; Birmingham et al., 2014). In order to exploit the envisaged potential, such devices will have to be “adaptive,” i.e., adjust “intelligently” and continuously their stimulation of neurons while monitoring effectiveness in real time. This is needed to counterbalance drift and intrinsic variability of the response to nerve stimulation through time, and to cope with patient-specific changes of conditions during daily life. Such a vision implies that BM must be also “precise,” i.e., allow for a finely tuned control of the nervous system by means of modulating neuronal excitability. Achieving this vision and conferring such a degree of “intelligence” to a miniaturized implantable device is a tremendous challenge. As pointed out above, significant technological progress has been made in artificially emulating neurons, synapses, and neuronal networks by low-power neuromimetic microelectronics. Beyond the capability to “speak the same language” made of nerve impulses and distributed computation, such neuromimetic architectures share with biological neuronal networks other properties, as online learning and reconfigurability based upon internal plastic changes (Qiao et al., 2015). The envisioned challenge toward brain repair and augmentation is to pair neuromorphic architectures with biological neurons *in vivo*, and set it to act as “chaperon or surrogate” of neuronal circuits to intelligently restore function. The neuromorphic devices will communicate bi-directionally (i.e., both receiving and sending nerve impulses) with biological neurons in the central or peripheral nervous system through advanced neural interfaces, enabling precise, and “near-physiological” tuning of neuronal activity within an “intelligent” adaptive closed-loop. In perspective, such approach could support a variety of bioelectronic and neuroprosthetic applications, independent of the physical nature of signals measured and stimuli delivered (i.e., electrical, chemical, etc.). It will set the context and the technological grounds for a true revolution toward “intelligent” neuroprosthetics and augmentation of brain function.

### Bibliometrics on neurobiohybrids research

The success of neurobiohybrids in neuroprosthetics will depend on community building, paralleling scientific-technological development, and directed to strenghtening of interactions, e.g., between neuroscientists, neurologists, and neurotechnologists and the communities working on brain-inspired computation and microdevices. We analyzed the development of such interactions in recent years by means of bibliometric analysis.

Bibliometrics was performed on publications related to neurobiohybrids to quantify interaction trends within the new interdisciplinary community. The details of the analysis procedure is provided in Section Methods. For the analysis, a total of 5320 journal articles and conference papers were carefully selected from three commercial scientific repositories (IEEE-Xplore, Thompson Reuters Web of Knowledge, and Elsevier's Scopus) through keyword searching for a window of 20 years (1995–2014). After careful selection, 3914 articles from 125 journals and 1406 papers from 93 conferences were taken into consideration for further analysis.

**Figure 4:**
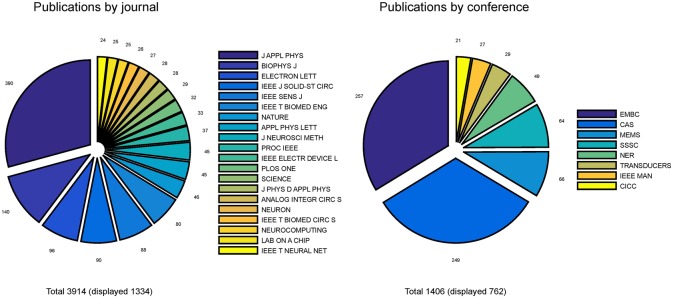
**Distribution of publications**. Distribution of journal articles **(left)** and conference papers **(right)** per year are reported as slices of pies. Only journals and conferences were considered with more than 20 articles and papers in the neurobiohybrid field.

Pie-charts in Figure 4 report the number of publications appeared in top 20 journals and top 8 conferences. It is interesting to note that the great majority of publications are mostly found in applied physics or engineering journals, with only a few appearances in multidisciplinary journals (e.g., Nature, Science and PLoS One) and even less in neuroscience journals (e.g., Neuron). From this first indications, it is tempting to conclude that the field is still very much biased toward engineering and physics communities rather than neuroscience communities. Additionally, it appears that only a few cutting edge publications have gained visibility to a wider community by publication on prestigious multidisciplinary journals. These results imply that the vast majority of researches in the neurobiohybrid field are not reaching the neuroscientific counterparts, which is a limiting factor for further development of this highly interdisciplinary field of research.

Also conference publications are dominated by engineering meetings. This can be in part explained as in neuroscience it is uncommon to publish results in conference papers, which are conversely well evaluated in the engineering environment. Again, efforts should be made to improve homogeneity of dissemination and to reach a wider audience.

**Figure 5:**
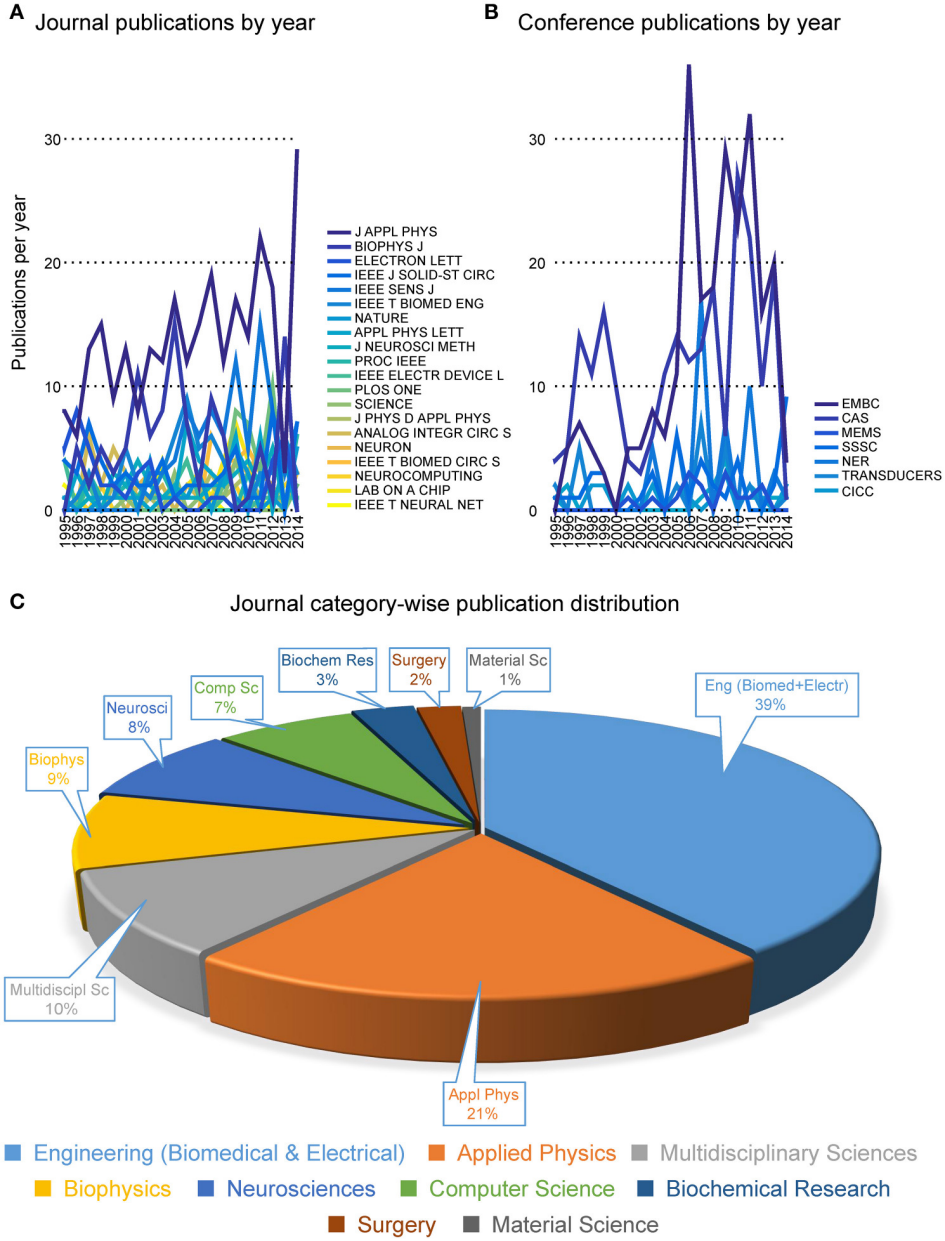
**Publication trends**. Distribution of the number of publications per year in journals **(A)** and conferences **(B)**. The journal category-wise publication distribution **(C)** shows dominating appearance of publications from the Neurobiohybrid field in journals belonging to the Engineering and Physical Sciences category, in comparison to the Health and Life Sciences category.

**Figure 6:**
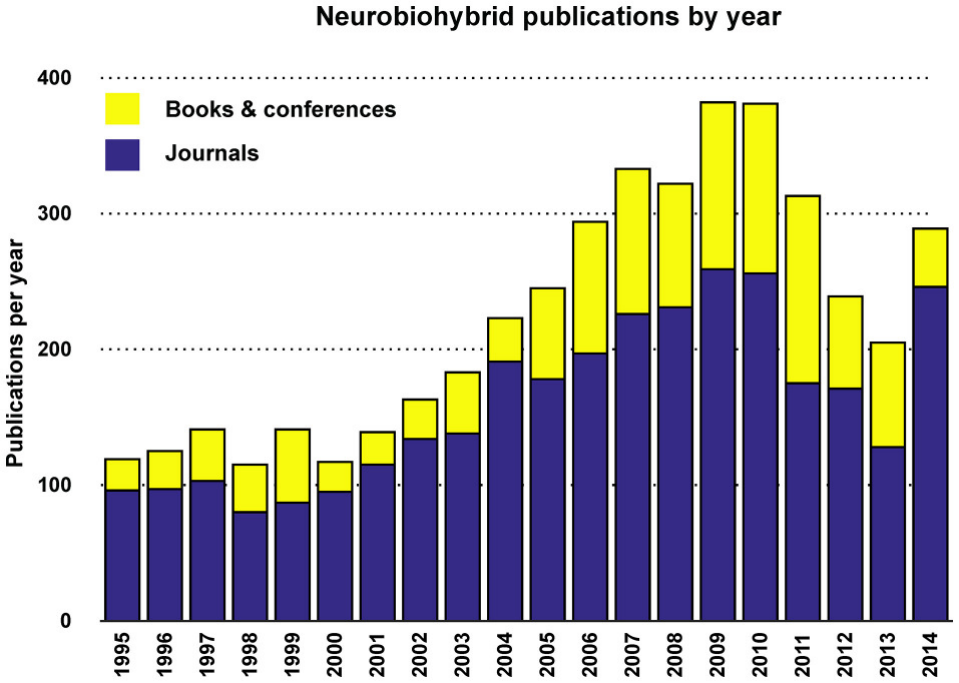
**Yearly total publications**. Total number of publications appeared in journals, conference proceedings, and books per year.

Judging from the trend of the number of publications per year (Figures 5, 6), a reasonable growth of the field can be appreciated, although comparable with other research fields.

**Figure 7:**
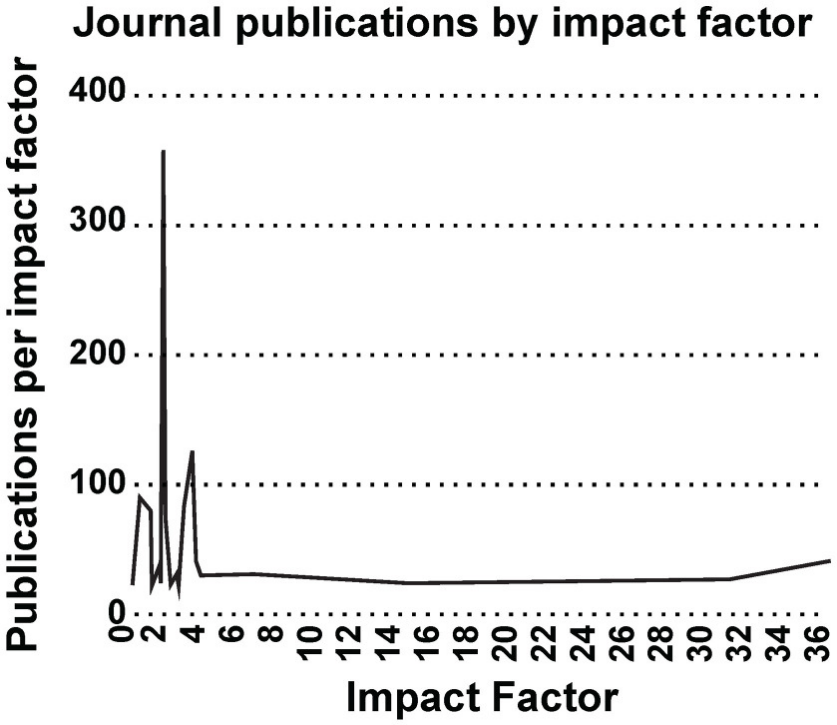
**Impact Factor distribution**. Impact factor distribution of publications in the neurobiohybrid field shows that majority of the publications appeared in journals with impact factor less than 5.

**Figure 8:**
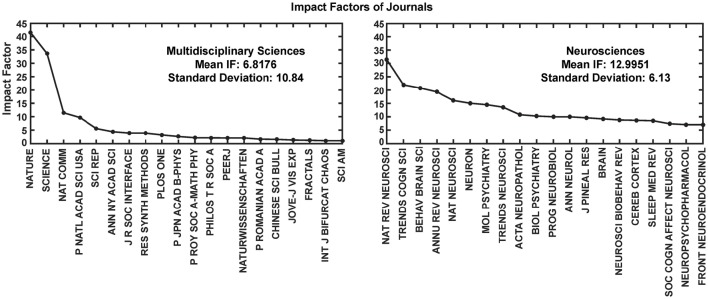
**Impact Factor comparison**. Two categories of journals were compared for the impact factors (IF) during the year 2014. These journals' IFs were calculated by Thomson Reuters and reported through Journal Citation Reports (https://jcr.incites.thomsonreuters.com/). Average IFs of the journals in the multidisciplinary sciences category (left) is almost half in comparison to the neurosciences category (right). For better representation only top 20 journals from each category are shown in the figure.

**Figure 9:**
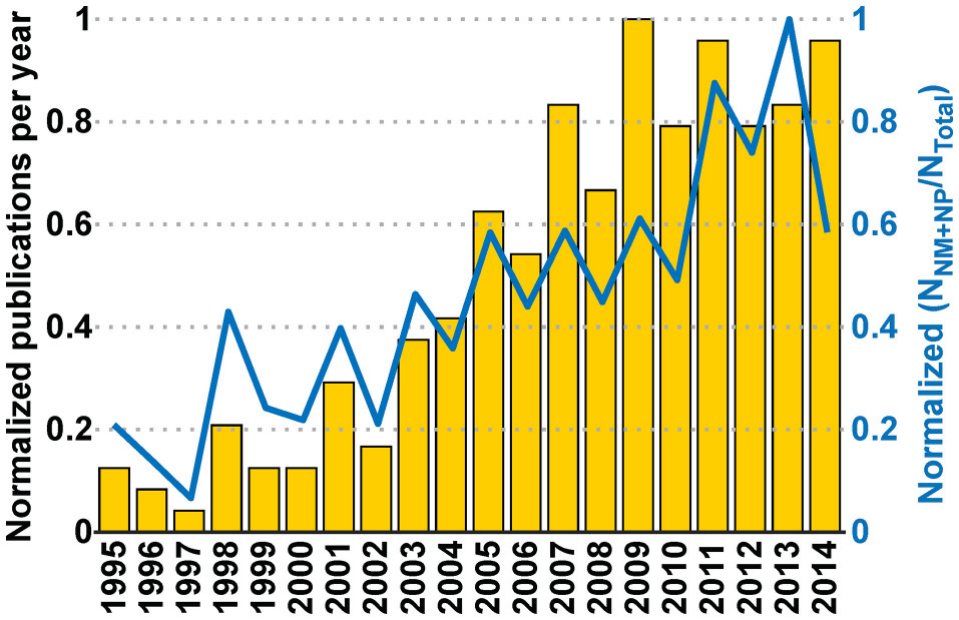
**Interaction between communities and its effect on the Neurobiohybird publications**. The bar plot (left Y-axis) shows the normalized publication frequencies calculated by extracting relevant publications through “interaction keywords”-based querying (see Section Analysis of Database) of the XML database. The increasing trend of the publication frequencies from two subcommunities (“Neuromimetics” and “Neuroprosthetics”) associated to the Neurobiohybrid field demonstrates a growing interaction between them. The interaction effect on the Neurobiohybrid publications is evident in the line plot (right Y-axis) which reports the yearly normalized ratios between subsets of publications resulting from the interactions (N_NM+NP_) and total number of journal publications in the Neurobiohybrid field (N_Total_).

Most interestingly, an analysis on the impact factor (IF) distribution (Figures 7, 8) reveals that most publications fall within a window below impact factor 5 with a high peak at around 2. While this may be acceptable within the engineering community, it is far below average with respect to publications in the neuroscience community. This discrepancy is playing against the building of a homogeneous community with equal career opportunities for engineers or physicist, on one side, and neuroscientists on the other side. Particularly, neuroscientists working in the neurobiohybrid field seem heavily penalized in terms of IF and will struggle in the competition with colleagues of other neuroscientific disciplines. Thus, in our view, the neurobiohybrid community should invest major efforts to improve dissemination efficacy, in particular, by increasing visibility of matured results through publications in interdisciplinary journals that can attract interest from a broader neuroscience community. In fact, as shown in Figure 5C, only 10% of the journal publications concerning neurobiohybrids appear in multidisciplinary journals and expanding this share will favor the communities' coalescence given the exemplary increasing interactions between the specialized subcommunities of Neuromimetics and Neuroprosthetics (Figure 9). Such efforts should be paralleled by organization of focused workshops and training initiatives in the neurobiohybrid field conceived in a way to attract interdisciplinary audience and to create a new generation of scientists with competences and skills spanning from neurotechnologies to neuromimetic systems and more classical neuroscience.

**Figure 10:**
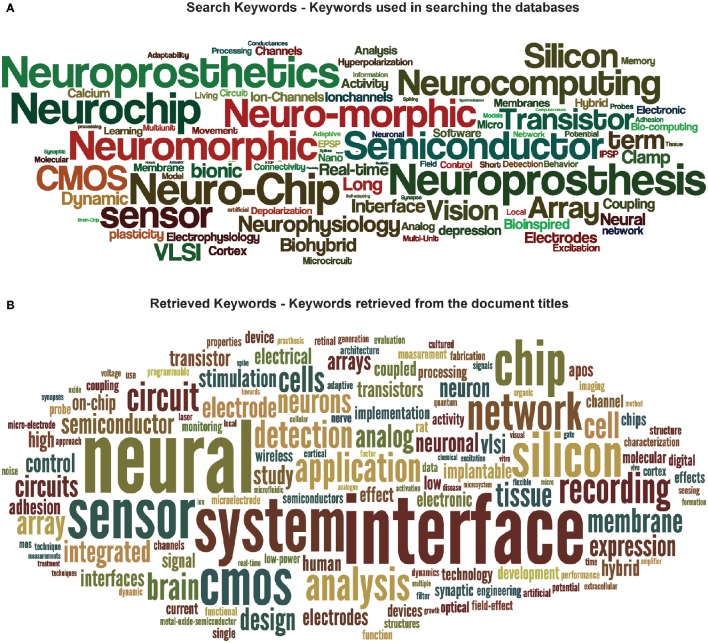
**Word cloud of keywords**. Word clouds of keywords used in searching the databases **(A)** and retrieved from the publication titles **(B)**. The size of a word in **(A)** denotes the number of times it was used with other search keywords in searching for publications in the database. On the other hand, in **(B)**, the size of a word represents its number of appearances in the publication titles.

Word clouds of keywords used for the bibliometric analysis are reported hereafter in Figure 10.

## Discussion

*Biohybrids*, that is, biohybrid systems where artificial devices and living organisms establish physical interactions with information exchange, will play a pivotal role in the future development of efficient, sustainable, and powerful information and communication technologies. A clear and well known example of biohybrid application supporting such expectations is represented by cardiac pacemakers, where information needed for restoring physiological heart pacing is provided by artificial rhythm generators through implanted electrodes. Noteworthy, closed-loop bi-directional interaction between organ and artificial pacemaker is seen as an important strategy to effectively restore function through a dynamic control and prevent cardiovascular pathologies (Occhetta et al., 2003). Biohybrids will represent an essential workbench to better investigate living organisms, to assess new principles of communication between natural and artificial world, and to develop novel generations of bio-inspired devices based on non-living matter. From the application perspective, they represent an innovative strategy to improve therapy of a variety of diseases through *in vivo* implants (Nicolelis and Lebedev, 2009). Overall, from a broad perspective, biohybrid technologies may replace artificial ones, leading to higher energy efficiency and performance gain while lowering environmental impact. Among biohybrids, *neurobiohybrids* are of paramount importance. After millions of years of evolution, the nervous system of living animals, and the human brain in particular, is endowed with unique abilities to cope with information processing in an energy effective, adaptable and robust manner, outperforming artificial devices when dealing with “real world” problems. Biohybrid systems of natural and artificial neurons implanted *in vivo* will be central to explore brain operational principles and, on the clinical side, to create novel generations of “intelligent” neuroprostheses.

### The paradigm shift

In our opinion, we are set to experience a true paradigm shift in neurobiohybrids research thanks to concomitant advances in three highly intertwined disciplines: *neurophysiology of brain microcircuits, neural interfaces*, and *neuromimetics* (that is, the creation of physical elements and circuits emulating living neurons and networks). For the first time, and thanks to recent development of physical elements with synaptic-like plasticity, a fascinating challenge is coming within reach: natural and hardware-based neuronal circuits could be integrated into new entities, operating *in vivo* through brain implants, and evolving together on the basis of shared plasticity and processing rules. To this endeavor, non-“von Neumann” brain-inspired architectures will have to be interfaced to their natural counterparts, the brain microcircuits. This will occur, at the physical level, through high-resolution, and bi-directional neural interfaces, and at the algorithmic level, by emulating in artificial architectures those processing rules that are key for the function of real biological brain networks. Noteworthy, however, neurobiohybrids can involve living networks at various levels of complexity and conditions, ranging from *in vitro* to *in vivo* systems and from “simple” nervous systems of invertebrates to the mammalian brain. Whatever the implementation strategy, the new hybrid systems will represent a technological platform with enormous potential not only for application in neuroscience and healthcare, as discussed below, but also in computer science and robotics. In fact, they will play a key role to understand operational principles of brain microcircuits and to developing new forms of brain-inspired computing devices more energy efficient and robust in dealing with real-world tasks.

From the theoretical point of view, the processing of information following the classical “von Neumann” digital computing paradigms is known to be less efficient compared to the biological counterparts, when dealing with ill-posed problems and noisy data. Though current computing technologies have reached speed and computational power figures that allows them to simulate parts of animal brains and behavior, the energy required by these systems grows exponentially with the increasing hierarchy of animal intelligence. The reason is that the biological brain is configured differently and the keys are the extremely high (~1015 synapses) connectivity between neurons in a network which offers highly parallel processing power as well as the fact that neurons are plastic and adaptive (i.e., memory dependent) signal processing and computing units. Yet, brain's most striking feature is that it is structured as an evolving system were synapses undergo “birth” and “death” as well as strengthening and weakening, reconfiguring neuronal connectivity in a self-organizing manner and allowing the networked population of neuronal processors to adapt motor and behavioral responses to the ever changing environmental inputs. Thus, by rearranging both the structural and functional topology, brain's neuronal circuits demonstrate unique evolvability, scalability, and adaptability properties that are unmatched by current computing devices. The challenge posed by neurobiohybrids research is to create networks where artificial elements overcome this deficiency by merging data storage and processing into single electronic devices, where topology can be reconfigured in a self-organizing manner, and to interface them to biological nervous systems. On-chip neuromorphic networks have recently emerged that may fulfill the purpose, and whose development is relying both on established microelectronic technologies (Indiveri et al., 2011; Indiveri and Liu, 2015) and novel approaches to emulate neuronal functions in single nanodevices (Indiveri et al., 2013; Gupta et al., 2016; Serb et al., 2016). Such artificial neural networks could provide the complexity, connectivity, and massive parallel information processing and thus mimic the performance of biological systems including their evolvability, self-organization, adaptability, and robustness. Following this vision, research on neurobiohybrids will on one hand enable significant progress toward novel “autonomous cognitive systems” while, on the other hand, it will promote the understanding of principles behind brain computation. The conception of brain-inspired implantable microdevices acting as “intelligent” neuroprostheses for brain rehabilitation and functional augmentation or as adaptive bioelectronic medicines will be the logical exploitation of such efforts toward clinical application.

In conclusion, we feel that we are at the beginning of a new era, where the fusion of neuromimetics and neurotechnologies for brain interfacing and creation of neurobiohybrids will lead to a new class of “smart” implantable systems with great potential for neuroscience and particularly for therapy of diseases of the nervous system. However, a process of community building is also necessary to reach the critical mass, which will have to overcome difficulties and hurdles. In particular, having a common and effective dissemination strategy, ensuring high visibility and career opportunities across all disciplines involved will be key aspect.

## Methods

The bibliometrics was performed following standard bibliometric methods as reported in Nathan et al. (2013). In short, a two-step method started with construction of an analysis database by searching and extracting information from three commercial scientific repositories using predefined search terms which was followed by analysis of the extracted publication data.

### Construction of search terms

As part of the Convergent Science Network's (CSN) road-mapping action, we had supplied questionnaires to experts belonging to the different communities mentioned above. Mining the answers provided to the question “Relevant state-of-the-art in your field of research” we formed a “keywords pool.” The unique keywords (*n* = 100) in that pool were then identified, combined and permuted to obtain the search terms (*N* = 862) which were used in querying the scientific repositories.

### Construction of analysis database

Three commercial scientific repositories were used to gather the publication information: (i) the IEEEXplore repository (http://ieeexplore.ieee.org/), (ii) the Thomson Reuters Web of Knowledge repository (http://apps.webofknowledge.com/), and (iii) Elsevier's Scopus (http://scopus.com/) repository. Out of the three, the IEEEXplore repository was used as source of articles published in IEEE journals and conferences, and the latter two were used for other journals and conferences.

Each of these repositories were searched for priorly defined keywords (or combinations of keywords, referred as “search terms” in the subsequent text, see Section Construction of Search Terms). The search domains were restricted to science, engineering, and life sciences for the Web of knowledge and Scopus repositories. But, the IEEEXplore repository was searched only science and engineering domain articles.

These repositories were queried using their built-in search engines which compared the search terms with the stored metadata (e.g., publication title, abstract, and author-defined keywords) corresponding to each indexed article. The metadata returned by the query as a result of a match with the given search term was appended to a predefined database created in EndNote reference management software (V7.4; Thompson Reuters, Philadelphia, USA; http://endnote.com/). At the end of the querying process, the Endnote database was exported to an extensible markup language (XML, http://www.w3.org/XML/) file (referred as analysis/XML database) and an automated in-house algorithm written in Matlab (R2015a; Mathworks Inc., Natick, USA, http://www.mathworks.com/) eliminated the redundant entries returned by overlapped queries in different repositories from the XML file. The tagged structure of XML file facilitated the application of Matlab's standard string-manipulation functions to extract the relevant information (e.g., article title, publication year, and title, etc.) from the metadata pertaining to each publication stored in the XML database. For each unique journals, its impact factor and category were manually retrieved from the Thompson Reuters Journal Citation Reports (JCR, https://jcr.incites.thomsonreuters.com/) and appended to the database.

### Analysis of database

The pre-processed metadata belonging to publication entries in the XML database were then analyzed to extract publication titles, unique journal and conference titles, and year of publication.

The following information were then extracted from the database:

1. Yearly publication frequency in journals or conferences (as reported in Figures 4–6), and journal category-wise publication distribution (Figure 5C; top 44 journals, from a descendingly ordered list of number of appeared articles, were categorized with a total number of 1200 articles and at least 10 articles in each journal during 1994–2014).
2. Impact factor distribution of the published articles belonging to the Neurobiohybrids (as reported in Figure 7) field. Also, comparison of impact factors of various journals belonging to the “Multidisciplinary Sciences” and “Neurosciences” category (see Figure 8).

In addition, two subcategories of seven keywords each (termed as “interaction keywords”) were defined by selecting popular keywords pertaining to two active subcommunities (Neuromimetics and Neuroprosthetics) in the Neurobiohybrid field. For the Neuromimetics, the selected keywords were: “neuromimetic,” “neuro-morphic,” “neuromorphic,” “neuro-chip,” “neurochip,” “neurocomputing,” and “sensor”; whereas for the Neuroprosthetics they were: “neuroprosthetic,” “neuroprosthesis,” “interface,” “brain machine interface,” “tissue,” “slice,” and “*in-vivo.”* The document titles, abstracts and author-provided keywords present in the XML database were searched for co-occurrence of at least one interaction keyword from each subcategories. The resulting publications along with their publication year were saved. The yearly publication frequency computed from the search results of the interaction keywords (see Figure 9) was used as a measure to determine interactions between the two subcommunities belonging to the Neurobiohybrids field.

Finally, the search terms and the retrieved keywords from the publication titles were represented as word clouds (see Figure 10) using a web-based free tool (wordle; http://www.wordle.net/) showing the frequencies of usage of each search keyword with another keyword (in case of search keywords cloud) and frequencies of occurrence of keywords in the publication titles (in case of retrieved keywords cloud).

## Author contributions

SV and MM performed the studies reported in the article and wrote the paper. Both authors have contributed to, seen and approved the final manuscript.

## Funding

Financial support from the 7th Framework Programme of the European Commission through “CSN II” (http://www.csnetwork.eu/, FP7-ICT-601167) and “RAMP” projects (http://www.rampproject.eu/, FP7-ICT-612058) are acknowledged.

### Conflict of interest statement

The authors declare that the research was conducted in the absence of any commercial or financial relationships that could be construed as a potential conflict of interest.
